# The effect of the video game Mindlight on anxiety symptoms in children with an Autism Spectrum Disorder

**DOI:** 10.1186/s12888-015-0522-x

**Published:** 2015-07-01

**Authors:** Lieke A. M. W. Wijnhoven, Daan H. M. Creemers, Rutger C. M. E. Engels, Isabela Granic

**Affiliations:** Behavioural Science Institute, Radboud University Nijmegen, P.O. Box 9104, 6500 HE Nijmegen, The Netherlands; GGZ Oost-Brabant, P.O. Box 3, 5427 ZG Boekel, The Netherlands; Trimbos Institute, Da Costakade 45, 3521 VS Utrecht, The Netherlands

**Keywords:** Treatment, Anxiety symptoms, ASD, Video game, Children

## Abstract

**Background:**

In the clinical setting, a large proportion of children with an autism spectrum disorder (ASD) experience anxiety symptoms. Because anxiety is an important cause of impairment for children with an ASD, it is necessary that effective anxiety interventions are implemented for these children. Recently, a serious game called Mindlight has been developed that is focused on decreasing anxiety in children. This approach is based on recent research suggesting that video games might be suitable as an intervention vehicle to enhance mental health in children. In the present study it will be investigated whether Mindlight is effective in decreasing (sub) clinical anxiety symptoms in children who are diagnosed with an ASD.

**Methods/Design:**

The present study involves a randomized controlled trial (RCT) with two conditions (experimental versus control), in which it is investigated whether Mindlight is effective in decreasing (sub) clinical anxiety symptoms in children with an ASD. For this study, children of 8–16 years old with a diagnosis of an ASD and (sub) clinical anxiety symptoms will be randomly assigned to the experimental (N = 60) or the control (N = 60) condition. Children in the experimental condition will play Mindlight for one hour per week, for six consecutive weeks. Children in the control condition will play the puzzle game Triple Town, also for one hour per week and for six consecutive weeks. All children will complete assessments at baseline, post-intervention and 3-months follow-up. Furthermore, parents and teachers will also complete assessments at the same time points. The primary outcome will be child report of anxiety symptoms. Secondary outcomes will be parent report of child anxiety, child/parent report of depressive symptoms, and parent/teacher report of social functioning and behavior problems.

**Discussion:**

This paper aims to describe a study that will examine the effect of the serious game Mindlight on (sub) clinical anxiety symptoms of children with an ASD in the age of 8–16 years old. It is expected that children in the experimental condition will show lower levels of anxiety symptoms at 3-months follow-up, compared to children in the control condition. If Mindlight turns out to be effective, it could be an important contribution to the already existing interventions for anxiety in children with an ASD. Mindlight could then be implemented as an evidence-based treatment for anxiety symptoms in children with an ASD in mental health institutes and special education schools.

**Trial registration:**

Dutch Trial Register NTR5069. Registered 20 April 2015.

## Background

In the clinical setting, a large proportion of children with an autism spectrum disorder (ASD) experience anxiety symptoms. Between 11 % and 84 % of all children with an ASD experience some degree of impairing anxiety [1]. More specifically, 21 % of the children with an ASD suffer from subclinical anxiety [2] and approximately 40 % of the children with an ASD meet the criteria of at least one anxiety disorder [3]. Some of the most frequently reported anxiety disorders and symptoms seen in children with an ASD are simple phobias, generalized anxiety disorder, separation anxiety disorder, obsessive-compulsive disorder and social phobia [1].

Moreover, anxiety is an underlying cause of several symptoms of ASD. For example, anxiety underlies or affects the stereotype and rigid behavior [4] and the problems in social functioning [5] that children with an ASD often show. Anxiety also underlies comorbid symptoms of children with an ASD, for example oppositional and aggressive behavior [6] and depressive symptoms [7]. Furthermore, anxiety in children with an ASD has a negative impact on adaptive functioning, daily living skills and relationships with peers, teachers and family [8–10]. Therefore, it is important that anxiety in children with an ASD is treated and prevented from further escalation.

Recognition of anxiety symptoms in children with an ASD is not new. In the original description of children with an ASD, Kanner [11] stated that a number of these children had “substantial anxiety problems”. Yet, the evaluation and treatment of anxiety in children with an ASD has only recently received empirical attention [1]. Many studies showed the effectiveness of adapted versions of cognitive behavioral therapy (CBT; *e.g.* [12]) or new interventions especially developed for children with an ASD (*e.g.* [13]), reasoning that the traditional form of CBT is not suitable for children with an ASD. On the other hand, a recent study of Van Steensel and Bögels [14] has shown that CBT is effective in reducing anxiety symptoms in children with an ASD, and that CBT is as effective for children with an ASD as for children without an ASD.

However, there are some important limitations to CBT for anxiety, both in general and specifically for children with an ASD. First, CBT largely consists of teaching children to become conscious of their negative thoughts, to evaluate these thoughts, and eventually to challenge them and formulate thoughts that are more accurate. These sessions have a face-to-face, verbal and cognitively complex character. Because children with an ASD have a cognitive and social impairment, they have difficulties with learning skills in these CBT-sessions and as a result they are often not intrinsically motivated for CBT [15]. Therefore, a greater focus on visual aids and structured sensory information is an important requirement in anxiety treatment for children with an ASD [15, 16]. Second, there is a large gap between the knowledge that children gain in CBT and the implementation and practice of this knowledge in daily life. Especially for children with ASD, frequent practice and exposure opportunities are important in anxiety interventions [1]. However, the exercises in CBT that do exist are mostly de-contextualized and do not fully represent the situations in which children experience their anxiety. A third limitation of CBT is limited access to care and long waiting lists to care that is accessible [17]. Finally, the low cost effectiveness is a limitation of CBT, which many mental health institutions experience as a barrier to treatment delivery. Therefore, it is important that new anxiety interventions are developed that can provide a solution for the above mentioned limitations.

Recently, it has been shown that video games have the potential to enhance mental health and well-being in children and adolescents [18, 17]. For example, Merry *et al.* [19] found that the video game ‘SPARX’ was effective in reducing depressive symptoms among adolescents in the age between 12–19 years old. They concluded that it was a potential alternative to usual care for adolescents with depressive symptoms in primary care settings and that it could be used to address some of the unmet demands for treatment. More recently, the serious game Mindlight (Playnice Institute) has been developed for the treatment of anxiety disorders in children. A recent study has tested the effect of Mindlight on anxiety symptoms in school children [20]. This study showed that both the anxiety of the children who played Mindlight as the anxiety of the children who played the control game significantly decreased over time. However, no studies to date have investigated the effect of a serious game on anxiety symptoms of children with an ASD.

Mindlight has the aim to tackle anxiety in children by using several treatment mechanisms. First of all, it uses exposure techniques, one of the most empirically-validated treatment components of CBT for anxious individuals [21]. During exposure, individuals are gradually exposed to the threatening cues. In this way, they are getting habituated to these cues and eventually they are getting more comfortable and less anxious when being exposed to them. Moreover, Mindlight uses neurofeedback mechanisms, which is effective in decreasing anxiety symptoms of children [22]. These mechanisms are focused on regulating arousal levels associated with anxiety through relaxation and concentration. Finally, attention bias modification is incorporated in Mindlight, which is a therapy mechanism in which children learn to (a) disattend to threatening cues and shifting attention away from those cues and to (b) focus on positive aspects of the environment in the service of relevant goals [23].

It is hypothesized that Mindlight has the potential to serve as an effective new intervention for children with ASD and comorbid (sub) clinical anxiety symptoms, and that it can overcome the limitations of CBT. First, it is known that children with ASD often feel a close affinity for technology and games, which means that the participating children are probably intrinsically motivated to play a game like Mindlight in therapy [24]. Moreover, it has been reported that computer based training could be an effective tool in treatment for children with ASD, due to its visual and structured character [15]. Mindlight uses visual aids and structured sensory information to a great extent, both for creating a ‘scary’ exposure environment and for teaching important treatment concepts. Furthermore, Mindlight includes frequent practice and exposure opportunities. Because Mindlight can be played repeatedly, with the difficulty level increasing as children become better players, there is a great deal of practice and exposure involved in playing this game. As a result, the gap between knowledge and behavior may be substantially decreased and effective cognitive and emotional coping skills can be automatized and possibly generalized with practice. Finally, therapy skills can be practiced at home, which means that children have an easier access to mental health care. In this way, the waiting lists can become shorter and the therapy costs can be decreased when implementing a game like Mindlight as therapy tool.

In the present study, the primary aim is to investigate whether Mindlight is effective in reducing (sub) clinical anxiety symptoms in children with an ASD. The secondary aim is to examine whether Mindlight is effective in reducing parent report of child anxiety, and the anxiety-related depressive symptoms, social functioning and behavior problems of the participating children. To investigate these aims, a multi method symptom assessment is used, including parent, teacher and child reports [1]. If Mindlight turns out to be effective for anxious children with an ASD, it could be considered as a new therapeutic intervention next to the already existing approaches for anxiety in children with an ASD.

## Methods

The study design will be reported in line with the CONSORT 2010 Statement for reporting parallel group randomized trials [25]. The medical ethics committee CMO Arnhem-Nijmegen in the Netherlands has given approval for the conduction of this study (NL50023.091.14). Moreover, the study is registered in the Dutch Trial Register for RCT’s (NTR5069).

### Design

The present study involves a randomized controlled trial (RCT) with two conditions (experimental versus control), in which it is investigated whether the new video game Mindlight is effective in treating (sub) clinical anxiety symptoms in children with an ASD. For this study, children in the age of 8–16 years old with a diagnosis of an ASD according to the Diagnostic and Statistical Manual of Mental Disorders 4th Edition – Text Revision (DSM-IV-TR; [26]) will be screened for anxiety symptoms. The children with (sub) clinical anxiety symptoms will be selected and approached for participation.

**Fig. 1:**
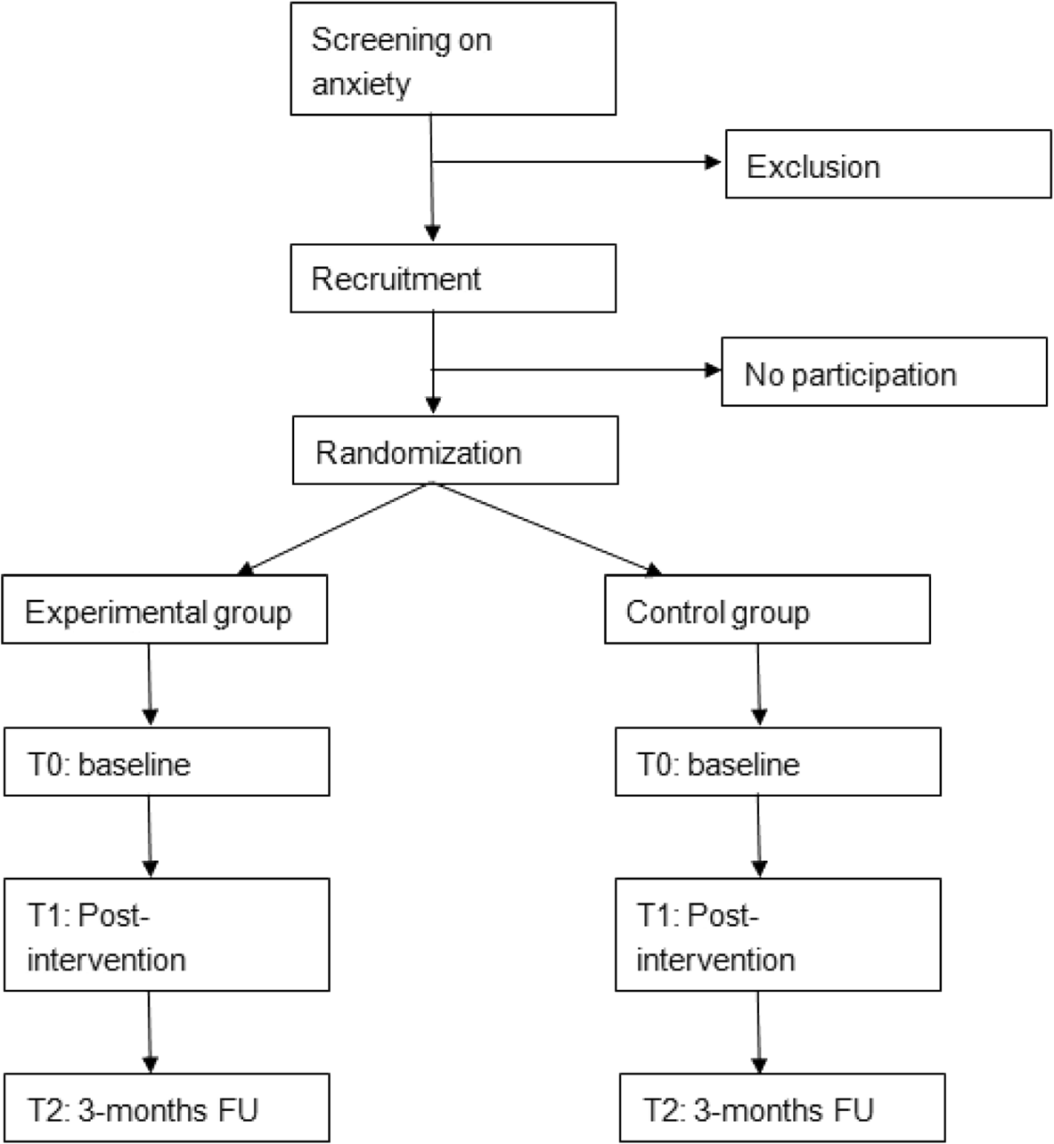
Flow diagram of recruitment, randomization and assessments. FU = Follow-up

After the selection and recruitment, children will be randomly assigned to the experimental or control condition. At baseline (T0), children, parents and teachers will fill in questionnaires. Moreover, parents will undergo a semi-structured interview (ADIS-P; [27]) to determine whether their child meets the criteria of one or more anxiety disorders. At post-intervention (T1) and at 3-months follow-up (T2), children, parents and teachers will fill in questionnaires again to evaluate the effect of Mindlight. At 3-months follow-up, parents will undergo the semi-structured interview again to test whether Mindlight also had an effect on the present anxiety disorder (s) in the participating children. Fig. 1 shows a schematic overview of the design in the present study.

### Participants’ eligibility

Children with an ASD (DSM-IV-TR: Autistic disorder, Asperger disorder, PDD-NOS; [26]) in the age between 8 to 16 years old will be assessed for eligibility by a screening. This screening consists of filling in an anxiety questionnaire by both children (SCAS-C; [28]) and parents (SCAS-P; [29]). When children have at least subclinical levels of anxiety, they are eligible for participation in the study. Moreover, they have to have sufficient knowledge of the Dutch language. Exclusion criteria are absence of parental permission and presence of prominent suicidal ideation or other severe psychiatric problems that need immediate treatment (*e.g.* severe trauma’s).

### Procedure

Contexts of recruitment are mental health institutes (*e.g.* GGZ Oost Brabant) and special education schools in the Netherlands. First, parents will receive a letter with information about the screening and the study. Moreover, children and parents will be asked to fill in the SCAS-C/P [28, 29]. When children have at least subclinical levels of anxiety and meet the other inclusion criteria, children and parents will be approached to participate in the study. If children and parents agree with participation, active written informed consent of the parents and the children who are above the age of 12 will be obtained.

After obtaining active written informed consent, children will be randomly allocated to the experimental or control condition. Children in the experimental condition will play Mindlight individually for one hour per week during 6 consecutive weeks at the recruitment location. Moreover, children may receive treatment as usual (TAU) parallel with Mindlight. TAU will mainly be offered by mental health institutes and might for example consist of psycho-education on ASD, play/drama therapy, parent guidance and/or medication. These types of TAU will be monitored and reported during the course of the study, and will be tested as potential confounders in the analyses. Children in the control condition will play the computer game Triple Town individually for one hour per week during 6 consecutive weeks at the recruitment location. Again, children may receive TAU parallel with the game. Moreover, they will have the opportunity to play Mindlight after the 3-months follow-up if this game turns out to be effective.

### Sample size

A priori power analysis was performed in G*Power 3.1 [30] to calculate the sample size that is required in the present study. It is expected that Mindlight is significantly more effective in reducing anxiety symptoms of the participating children than Triple Town. Previous studies on the effect of already existing modified versions of CBT on anxiety among children with an ASD reported moderate to large effect sizes on anxiety symptoms (*e.g.* [31, 12, 32]). It is assumed that the implementation of Mindlight in clinical practice is worth consideration, when the effect of Mindlight is at least equal to the effect of the present anxiety treatments for children with an ASD. Therefore, the power calculation is conducted with a moderate expected effect size (f = .25) of condition (experimental or control condition) at 3-months follow-up. Moreover, it was expected that the Type I error was .05 and that the Type II error was .20 (power is .80). When computing these assumptions, it was calculated that a sample of 86 participants would be satisfactory in the present study. Eventually, the sample size was increased by 40 % to compensate for loss to follow-up (estimated at 20 %) and possible loss of power due to potential clustering of data in case of group formation (estimated at 20 %), resulting in 120 participants (60 in experimental condition and 60 in control condition).

### Intervention

The intervention that will be investigated is called Mindlight. This is a video game aimed at children of 8–16 years old and is based on principles of cognitive-behavioural therapy (CBT) and neurofeedback, which are evidence-based interventions for anxiety-disordered children and adults. Briefly, the premise of the game is that Little Arthur is left on the doorstep of a scary mansion by his parents. Arthur must learn to use his own inner strength to overcome his greatest fears so the shadows in the house can hold no power over him. He can accomplish this by using his Mindlight, a light bubble that can shine on the surroundings and that can be controlled by his own inner strength. This ‘inner strength’ will be measured by a neurofeedback headset (the ‘MindWave’; Neurosky USA; [33]), which children will put on when they are going to play Mindlight. This headset records EEG using dry sensor technology, which consists of an active and reference electrode. The signals that are measured will be filtered on Delta, Theta, Alpha and Beta waves. In Mindlight, especially the Alpha and Beta waves will be used for real time feedback. Research has shown that the Mindwave headset has good reliability and validity [33], and that it can be used in research with children who have a developmental disorder (*e.g.* ADHD; [34]).

**Fig. 2:**
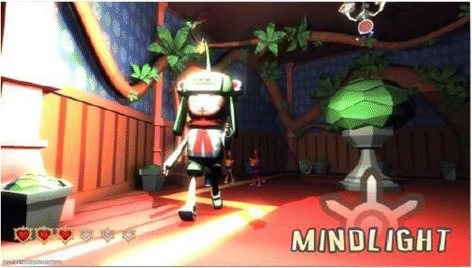
Relaxation mechanic Mindlight: remain calm to enlighten environment

In Mindlight, the Alpha and Beta waves will be used in several ways. First of all, the recorded Alpha waves reflect the degree of relaxation of the child. This feature is used in the exposure techniques (CBT) that are embedded in the game (see also Fig. 2): when the child sees threatening stimuli (*e.g.* monsters) several times during the game and learns to maintain calm when facing them, the child eventually gets habituated to them and can gain points more easily.

Furthermore, the recorded Beta waves reflect the degree of concentration and the allocation of attention of the player. Focused concentration allows the player to solve attention bias modification (ABM) puzzles. ABM is a training protocol that has its roots in CBT and that is based on the idea that distorted cognitions, particularly attentional biases characterized by hyper attention towards potential threats, play a role in the pathogenesis of childhood anxiety [22]. ABM has been shown to reliably reduce anxiety by retraining the attentional system to focus on positive stimuli [35]. Mindlight uses this principle in the ABM-puzzles, by rewarding children for focusing on positive aspects of the environment (measured by the neurofeedback device). More specifically, they learn to move towards, and quickly respond to, positive stimuli (*e.g.*, portraits of happy faces) and disattend to, or shift attention away from, negative stimuli (*e.g.*, mean faces, threatening animals).

To minimize the chance of finding placebo-effects of Mindlight, children in the control condition will also receive a computer game. In this way, the amount of attention that children in the experimental condition and children in the control condition receive is equal, and the effects that may be found can uniquely be ascribed to the game itself. The computer game that the children in the control condition are going to play is called ‘Triple Town’. In this puzzle game, the player has to build a city. The bigger the city you build, the more points you can receive. To accomplish this, you have to combine elements (*e.g.* trees and houses) in a strategical way. Moreover, you have to block bears that try to hinder you in building the city. In this way, the child learns to think strategically in order to overcome challenges. Moreover, children learn to keep a goal and to persevere in order to reach this goal. However, the game is not specifically focused at reducing anxiety levels, which makes ‘Triple Town’ a suitable game for the control condition.

The gaming sessions will be led by qualified therapists, or by master students who are supervised by qualified therapists. In session 1, the therapist starts with psycho-education on anxiety. After that, the anxiety of the child will be discussed, the therapist will explain the game and eventually the therapist will clarify that this game is focused on decreasing the anxiety of the child. Then, the child will play the game for approximately 40 min. After playing the game, the therapist will ask the following standardized questions: 1) How did the gaming go today? 2) What did you find difficult/What did you find easy? 3) What did you learn in the game? 4) Could you apply and practice the skills you have learned in scary or difficult situations in daily life? In session 2–6, the therapist will start the session with discussing the previous week and the skills the child has practiced at home. When the child mentions that he has practiced the skills in a scary or difficult situation, this will be reinforced by the therapist. In this way, the therapist does not add explicit therapeutic elements to the gaming sessions, but children do get stimulated to think about their anxiety and the way they can apply and practice the skills they have learned in the game in daily life.

### Study outcome measures

Table 1 shows an overview of the different time points, the questionnaires that were filled in on each time point and the informants that were involved.

**Table 1:** Overview of assessments

	Screening	T0	T1	T2
Child				
Anxiety (SCAS-C)	χ	χ	χ	χ
Depression (CDI 2)		χ	χ	χ
Therapeutic expectancies (PETS)		χ		
Parent*				
Anxiety (SCAS-P)	χ	χ	χ	χ
Anxiety disorders (ADIS-P)		χ		χ
Depression (CDI 2:P)		χ	χ	χ
Social functioning (VISK)		χ	χ	χ
Behavior problems (SDQ)		χ	χ	χ
Therapeutic expectancies (PETS)		χ		
Teacher				
Social functioning (VISK)		χ	χ	χ
Behavior problems (SDQ)		χ	χ	χ

*The primary caregiver of the child will fill in the questionnaires. The ADIS-P will be conducted with both parents (if possible)

### Screening measures

To test their eligibility, children will be screened on anxiety symptoms using the SCAS-C for child report and the SCAS-P for parent report [28, 29]. Children are eligible for participation when the child and/or the parent report the presence of subclinical child anxiety. Moreover, demographical questions (*e.g.* sex, age, educational level) will be asked to both children and parents. Finally, some questions about the child’s gaming behavior (*e.g.* ‘How many hours per week do you game?’) will be asked.

### Primary outcome measure

*Anxiety symptoms* will be measured with the Dutch translation of the Spence Children’s Anxiety Scale (SCAS; [28]). The SCAS consists of 44 items (*e.g.* ‘I am afraid when I have to sleep alone’, ‘I worry about things’) on a 4-point scale, ranging from ‘never’ to ‘always’. Scores on items ranged from 0 to 3, with higher scores indicating more anxiety symptoms. Moreover, the scale consists of six subscales that are in line with the different anxiety disorders that are described in the DSM-IV: panic/agora phobia, separation anxiety, social phobia, generalized anxiety, obsessive compulsive anxiety and anxiety for physical injury. The SCAS has a high validity and reliability [36, 37].

### Secondary outcome measures

*Anxiety of the child according to the parents* will be measured with the Dutch translation of the Spence Child Anxiety Scale for Parents (SCAS-P; [29]). The SCAS-P consists of 38 items on a 4-point scale ranging from 0 (never) to 3 (always). The items of the SCAS-P were formulated as closely as possible to the corresponding item of the child version of the SCAS. Only items referring to an internal state (*e.g.* item 4: ‘I feel afraid’) were rephrased into observable behaviour for parents (*e.g.* ‘My child complains of feeling afraid’). The SCAS-P consists of the same six subscales as the child version. The SCAS-P has a good reliability and validity [38].

*The presence of anxiety disorders according to the parents* will be assessed with the Dutch translation of the Anxiety Disorders Interview Schedule for DSM-IV, Parent version (ADIS-P; [27]). This is a semi-structured diagnostic parent interview that can be used to diagnose anxiety disorders in children of 7–17 years old. The interview will be administered by a qualified therapist or by a master student under supervision of a qualified therapist. In this study, the presence of the following DSM-IV anxiety disorders will be examined: separation anxiety disorder, social phobia, specific phobia, panic disorder, agora phobia and generalised anxiety disorder. The interview consists of standardized questions, with ‘yes’, ‘no’ and ‘different’ as possible answers. At the end of the interview, the interviewer has to give his/her clinical judgement about the severity of every disorder. On basis of this judgement, the interviewer will make a definitive decision about the presence (yes/no) of the different anxiety disorders. The ADIS-P has a strong reliability and validity [27].

*Depressive symptoms* will be measured using the Dutch translation of the Child Depression Inventory 2 (CDI 2; [39]). The CDI 2 consists of 28 items measured on a 3-point scale ranging from 0 (depressive symptom is absent) to 2 (depressive symptom is always present) (*e.g.*, ‘I don’t feel alone’ = 0, ‘I often feel alone’ = 1, ‘I always feel alone’ = 2; ‘Sometimes I’m sad’ = 0, ‘I’m often sad’ = 1, ‘I’m always sad’ = 2). The children have to choose the answer that is most in accordance with their own thoughts and feelings. The validity and reliability of the Dutch CDI 2 (:P) is still being investigated, but it already has been shown that the original (American) version of the CDI 2 (:P) [40] has a good reliability, internal consistency and convergent validity [41].

*Depression according to the parents* will be measured with the Dutch translation of the Child Depression Inventory 2 for parents (CDI 2:P; [39]) will be used to measure parental assessment of depressive symptoms of their child. The CDI 2:P consists of 17 items measured on a 4-point scale ranging from 0 (not at all), 1 (some of the time), 2 (often), or 3 (most of the time) (*e.g.* ‘My child looks sad’; ‘My child seems lonely’). The parent has to assess to which extent the items are in accordance with their child’s thoughts and feelings.

*Social functioning according to the parents and teacher* will be measured with the ‘Vragenlijst voor Inventarisatie van Sociaal gedrag van Kinderen’ (VISK), a Dutch translation of the Children’s Social Behaviour Questionnaire (CSBQ; Luteijn [42]). The VISK consists of 49 items measured on a 3-point scale (0 = not applicable, 1 = sometimes applicable, 2 = often applicable). The items are divided over six problem scales: being not well tailored to social situations; limited tendency to engage in social interactions; orientation problems in time, space and place; not understanding social information; stereotype behavior; and anxiety for and resistance against changes. Hartman and colleagues [43] reported that the VISK has a good validity and reliability.

*Internalizing and externalizing behaviour problems according to the parents and teacher* will be measured with the Dutch translation of the Strengths and Difficulties Questionnaire (SDQ; [44]). The SDQ consists of 25 items, measured on a 3-point scale (0 = not true, 1 = a little bit true, 2 = certainly true). Moreover, an impact supplement can be completed, by which the impact of the behavior problems on daily functioning of the child and its family can be assessed. The items of the SDQ are divided over the following scales: emotional problems, behavior problems, hyperactivity/ attention problems, problems with peers and social behavior. There are separate forms for parents and teachers. The SDQ has a sufficient reliability and validity [45].

*Treatment expectancies according to child and parents* will be measured using the Dutch translation of the Parent Expectancies for Therapy Scale (PETS; [46]). The PETS has a parent and child version, and each version consists of seven items measured on a 6-point scale ranging from 1 (I totally disagree) to 6 (I totally agree). The items of the PETS are divided over the following subscales: credibility, child improvement and parent involvement. The PETS has a good validity and reliability [47].

### Data analysis/statistical analysis

Following the intention-to-treat principle [48], all children randomized to a condition will be included in the analyses to test the study objectives. Multiple imputations will be used for missing observations at post-intervention and 3-month follow-up. Reporting of the results of the study will be in accordance with the CONSORT 2010 Statement [25].

To investigate the differences in the development of anxiety symptoms between children in the experimental condition and children in the control condition, a 3 (within-subjects: pre, post, follow-up) by 2 (condition: experimental vs. control) ANOVA (repeated measures) will be conducted, with anxiety symptoms (child report) as the dependent variable. The direct effect of Mindlight on parent report of child anxiety, parent/child report of depressive symptoms, and parent/teacher report of social functioning and behavior problems will be investigated in the same way. Furthermore, remission rates of the anxiety disorders that were diagnosed at baseline (with ADIS-P) will be calculated at 3-months follow-up, and Chi-square (*χ*^2^) tests will be conducted to compare remission rates between the experimental and control group. In the above mentioned analysis plan it is assumed that the children will play the game individually and that therefore the data will not be clustered. However, in case of clustered data due to the formation of groups in which several children play the game in the same room, the analyses will be conducted in MPLUS 6.11 [49].

Finally, possible baseline differences between the two conditions in demographic variables (*e.g.* age, sex, educational level), anxiety symptoms, gaming behaviour and treatment expectancies will be checked. Moreover, possible differences in TAU between the experimental and control condition will be checked at all time points. Variables that show different distributions between the two groups will be entered as confounders in all models testing the effectiveness of the intervention.

## Discussion

The present study protocol gives an overview of a study design for a randomized controlled trial testing the effect of the serious game Mindlight in decreasing anxiety symptoms of children with an ASD. The primary aim is to investigate whether Mindlight is effective in reducing (sub) clinical anxiety symptoms of children with an ASD in the age of 8–16 years old. The secondary aims are to investigate whether Mindlight is effective in reducing parent report of child anxiety and the anxiety-related problems in social functioning, depressive symptoms and behavior problems of children with an ASD. It is expected that Mindlight is effective in reducing anxiety symptoms of the children in the experimental condition, compared to the children in the control condition that play Triple Town. Furthermore, it is expected that Mindlight is more effective than Triple Town in reducing parent report of child anxiety, parent/child report of depressive symptoms and parent/teacher report of social functioning and behavior problems.

### Strengths and limitations

The present study design has a few strengths and limitations. A strength is that it is the first study investigating the effect of a serious game in a clinical context with children who are diagnosed with an ASD and who have comorbid (sub) clinical anxiety symptoms, which could lead to a new way of treating anxiety in children with an ASD. An additional strength is that children with an ASD often feel a close affinity for technology and games [24, 15], and that the participating children are probably intrinsically motivated to play a game like Mindlight in therapy. Furthermore, Mindlight includes frequent practice, exposure opportunities, visual aids and structured sensory information, which all stimulate the automatization and the generalization of skills to daily life in the participating children. Another strength is that this study may lead to the implementation of Mindlight in mental health institutes, which may result in an easier access to mental health care, shorter waiting lists and lower therapy costs. Finally, this study has multiple outcome measures, which makes it possible to investigate other direct effects of Mindlight on anxiety-related symptoms (*e.g.* depressive symptoms) of the participating children.

A limitation of the present study design is that the children in the control condition might have played Triple Town already before the start of the study. This may have a positive (*e.g.* more practice) or negative (*r* boredom) influence on the effect of the game. Moreover, only short-term effects (3-months follow-up) of Mindlight will be investigated. In this way, no conclusions can be drawn about the long-term effects of Mindlight on anxiety symptoms of the participating children. Finally, there are no standardized protocols for offering and implementing a video game in a clinical therapy session. This implies that the best form of implementation still needs to be discovered and improved by experience.

### Implications for practice

Anxiety symptoms are highly common in children with an ASD. Still, treatment on anxiety in children with an ASD only recently has received some empirical attention. This may be caused by the fact that anxiety is often underdiagnosed in children with an ASD [1]. In this way, anxiety treatment for children with an ASD is not common and the development of evidence-based anxiety treatments has not been focused upon until recently. By developing and investigating new anxiety treatments for children with an ASD, these may be more frequently offered in mental health institutes in the future. Moreover, if Mindlight turns out to be effective for anxious children with an ASD, it could be considered as a good and suitable therapeutic alternative to the already existing interventions for anxiety in children with an ASD. Mindlight could then be implemented as an evidence-based treatment for children with an ASD in mental health institutes and special education schools.

## Conclusion

This paper aimed to describe a study that will investigate the effect of the serious game Mindlight on (sub) clinical anxiety symptoms of children with an autism spectrum disorder in the age of 8–16 years old. It is expected that children in the experimental condition will show lower levels of anxiety symptoms at 3-months follow-up, compared to children in the control condition. If Mindlight turns out to be effective, this could provide a significant contribution to the evidence-based treatment of anxiety in children with an ASD.
